# Editorial: Neurological and psychiatric disorders: The role of clathrin-mediated endocytosis (CME) and related intracellular trafficking

**DOI:** 10.3389/fncel.2023.1165675

**Published:** 2023-03-10

**Authors:** Isabella Zanella, James A. Daniel, Daniela Zizioli

**Affiliations:** ^1^Department of Molecular and Translational Medicine, University of Brescia, Brescia, Italy; ^2^Clinical Chemistry Laboratory, Section of Cytogenetics and Molecular Genetics, Spedali Civili di Brescia, Brescia, Italy; ^3^Department of Molecular Neurobiology, Max Planck Institute for Multidisciplinary Sciences, Göttingen, Germany

**Keywords:** clathrin-mediated endocytosis (CME), clathrin coated vesicles, intracellular trafficking, neurological disorders, psychiatric disorders

Clathrin-mediated endocytosis (CME) is a cellular fundamental process in which cargo molecules are internalized from the cell surface and trafficked into vesicles within the cell. In keeping with cargo diversity, CME has different physiological functions, from molecule uptake, receptor internalization, signal transduction, synaptic vesicle recycling (SVR). Clathrin is also essential in intracellular trafficking of membranes of the endoplasmic reticulum (ER), ER-Golgi intermediate compartment (ERGIC), trans Golgi Network (TGN), endosomes, lysosomes, and plasma membrane.

CME is particularly essential for neural functions. Evidence of its crucial functions is that its dysregulations are common hallmarks of several neurological diseases.

The aim of this Issue was to provide a comprehensive overview of the role of CME and related intracellular trafficking in neurological and psychiatric diseases. Several researchers contributed interesting point of views, focusing on diseases caused by mutations in proteins involved in these pathways and reviewing different aspects of their dysregulation.

In the CME process two key endocytic proteins play important roles: clathrin and dynamin. Small molecules inhibitors can interact with both proteins resulting in the inhibition of the CME process. This inhibition might be used as a potential therapeutic target on neurological and psychiatric disorders. Dynamin 2 (DNM2) belongs to the dynamin family of large GTPases that catalyze membrane constriction and fission during multiple cellular processes including endocytosis. DNM2 consists of an N-terminal GTPase domain; a middle domain that mediates dimerization; a pleckstrin homology domain (PHD) that binds to phosphatidylinositol 4,5-biphosphate (PIP2); a GTPase effector domain (GED) regulating GTPase activity and a C-terminal proline/arginine-rich domain (PRD) that interacts with proteins containing the Src homology 3 domain. The middle and the GED domains form the stalk mediating DNM2 self-assembly. Assembly is regulated through conformational changes induced by PIP2 binding resulting in a closed-to-open conformational switch. On the other hand, clathrin has a complicated structure and its activity requires the assembly of a macromolecular complex, a triskelion. Since long time clathrin have been well-characterized (Pearse, 1975), having a central role in CME, mitosis and SVR. In this Issue, Prichard et al. well-described the important role of clathrin and suggested CME as one of the major mechanisms for SVR in neurological diseases such as Parkinson's disease (PD), epilepsy, schizophrenia. In the area of these neurological diseases drug development has focused primarily on postsynaptic targets and G-protein coupled receptors, leaving the investigation of endocytic machinery as potential drug targets an unexplored but promising field. Recently the discovery of molecules able to inhibit CME highlighted its potential medical importance. Generic approaches to CME inhibition include environmental stimuli which acts as general cellular perturbant such as cytosolic acidification, potassium depletion, hypertonic treatment, and small molecules as monodansylcadaverine, chloroquine, ikarugamycin.

DNM2 mutations mainly occurs in its PHD domain: those causing centronuclear myopathy (CNM) disrupt the closed conformation, enhancing self-assembly and GTPase activity without affecting PIP2 binding (gain of function), while those associated with Charcot Marie Tooth disease (CMT) inhibit PIP2 binding and PIP2 stimulated GTPase activity, resulting in self-assembly inhibition (loss of function). Tassin et al. elegantly showed that one CMT-causing mutation, lacking residues ^555^DEE^557^ (ΔDEE), did not alter PIP2 binding and conferred resistance to *in vitro* disassembly, stabilizing DNM2 polymers, similarly to CNM-causing mutations. On the converse, the mutation did not induce DNM2 oligomerization in living cells. The authors also showed that the mutation, probably acting on the protein conformation, enhanced tyrosine phosphorylation in cells expressing c-Src, compared to wild-type DNM2 or protein harboring CNM- or CMT-linked known mutations. The authors concluded that molecular mechanisms underlying DNM2 mutations should be further deepened.

Snead and Gowrishankar focused their study on Mitogen-Activated Protein Kinase 8 Interacting Protein 3 (MAPK8IP3), a putative adaptor protein that in neurons is believed to link lysosomes to dynein and kinesin motors during retrograde axonal transport. In all studied animal models, the loss of this protein results in axonal lysosome accumulation. *De novo* variants in MAPK8IP3 have been found in children with neurodevelopmental disorders and intellectual disability. A study in *C. elegans* of some of these variants showed that while two of them resulted in axonal lysosome accumulation, further variants affected worm locomotion (Platzer et al., 2019), suggesting that MAPK8IP3 may be involved in further cellular processes. With this aim, the authors observed the effects of MAPK8IP3 knocking out in neurons derived from induced pluripotent stem cells and found that loss of MAPK8IP3 resulted in axonal lysosome accumulation, as previously observed. More interestingly, they also observed that the cells did not reduce lysosomal proteolytic activity but exhibited defective endocytic activity, revealing a potential new cellular role for the protein.

In their comprehensive review, Deo and Redpath extensively described the endocytic regulation of serotonin (5-hydroxytryptophan, 5-HT) transporter (SERT) and 5-HT receptors, particularly focusing on 5-HT_1A_ and 5-HT_2A_ receptors and their roles in 5-HT extracellular levels and depression or anxiety. The authors also detailed how selective serotonin reuptake inhibitors (SSRI) modulate 5-HT signaling through endocytosis. Through a detailed description of genetic, transcriptomic and proteomic changes associated with depression and anxiety, they provided an in-depth overview of endocytic dysregulation in those psychiatric conditions. Finally, the authors proposed an interesting model of how genetic, transcriptomic and proteomic changes in patients with depression and anxiety could perturb endocytosis of 5-HT and SERT.

Finally, Smith et al. showed that α-synuclein, a central component of the characteristic cell death in PD, can propagate in neurons and that a combination of environmental and genetic factors are thought to contribute to the development of PD. Different studies suggested that α-synuclein is internalized *via* CME beginning with α-synuclein accumulating on the plasma membrane. Inhibition of α-synuclein aggregation is the new model of treatment and eventual prevention of neurodegeneration in PD patients. The analysis of different uptake and clearance system highlights the importance for further understanding of α-synuclein trafficking and its biology. Protein, uptake, trafficking and clearance is a vital pathway to understand PD pathogenesis and is a key target for therapeutic intervention.

In conclusion, many questions are still open to better understand the role of CME in neurological and psychiatric disorders. This Research Topic of reviews and articles raises crucial issues to be considered in this fascinating research field.

## Author contributions

All authors contributed to manuscript preparation and approved it for publication.
